# Systemic Consequences of Chronic Ethanol Intake: From Microbiome Shifts to Metabolic Impairment

**DOI:** 10.1002/cph4.70132

**Published:** 2026-03-26

**Authors:** Muni Swamy Ganjayi, Thomas A. Krauss, Gage E. Demster, Sehyung Park, Garrett B. Anspach, Sarah R. Anthony, Shaohua Wang, Michael Tranter, Robert N. Helsley, Cory W. Baumann

**Affiliations:** ^1^ Department of Biomedical Sciences, Heritage College of Osteopathic Medicine Ohio University Athens Ohio USA; ^2^ Ohio Musculoskeletal and Neurological Institute Ohio University Athens Ohio USA; ^3^ Internal Medicine, Saha Cardiovascular Research Center, Barnstable Brown Diabetes and Obesity Center, Markey Cancer Center University of Kentucky Lexington Kentucky USA; ^4^ Department of Molecular Medicine and Therapeutics, Davis Heart and Lung Research Institute The Ohio State University Columbus Ohio USA; ^5^ Infectious and Tropical Disease Institute Ohio University Athens Ohio USA; ^6^ Institute of Molecular Medicine and Aging Ohio University Athens Ohio USA

**Keywords:** alcohol use disorder, dyslipidemia, gut‐liver axis, tissue dysfunction

## Abstract

Chronic ethanol (EtOH) consumption is a major contributor to multi‐organ dysfunction, yet its systemic effects remain incompletely understood. To address this, we utilized a physiologically relevant long‐term mouse model, administering 20% EtOH in drinking water for 60 weeks, to investigate the integrated consequences of chronic exposure. EtOH‐consuming mice (0.4–0.5 mL/day) exhibited > 30% reductions in chow and fluid intake, resulting in a 12% decrease in total caloric intake compared to controls (*p* < 0.001). Body mass remained similar until Week 52, after which EtOH‐treated mice had lower body mass due to reductions in both lean and fat mass (*p* ≤ 0.004). Functional assessments revealed impaired treadmill endurance (−17%) and grip strength (−11%) (*p* ≤ 0.037), while motor coordination remained unaffected (*p* = 0.203). Chronic EtOH exposure significantly altered gut microbiota composition, reducing *Lactobacillus* and enriching *Faecalibaculum*, *Clostridium*, and *Bifidobacterium* at the genus level. These changes were accompanied by marked depletion of short‐chain fatty acids (*p* ≤ 0.05). Indirect markers of gut permeability (serum LPS & zonulin) and liver injury (serum ALT & AST, hepatic amyloid content) were elevated, alongside increased total cholesterol and > 62% upregulation of hepatic TNFα, IL‐6 & serum amyloid A (*p* ≤ 0.046). EtOH also induced dyslipidemia and glucose intolerance (*p* ≤ 0.041), although transcriptomic changes in white adipose tissue were minimal despite elevated free fatty acids. In conclusion, chronic EtOH consumption disrupts energy balance, compromises gut barrier integrity, and impairs hepatic metabolism, collectively driving systemic and metabolic dysfunction. These findings underscore the gut‐liver axis as a key mediator of EtOH‐induced pathology and highlight the gut microbiome as a promising therapeutic target.

## Graphical abstract

Chronic ethanol intake disrupts whole‐body homeostasis by reducing energy intake and body mass, impairing physical function, and reshaping the gut microbiome. Microbiota shifts and depletion of short‐chain fatty acids increase gut permeability and drive hepatic inflammation, dyslipidemia, and glucose intolerance. Together, these effects identify the gut–liver axis as a central mediator of ethanol‐induced metabolic dysfunction.

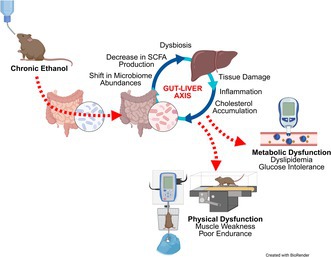

## 1 Introduction

Ethanol (EtOH) has widespread and complex effects on mammalian physiology, influencing nearly every tissue and organ system. Its impact ranges from acute modulation of cellular signaling pathways to long‐term disruptions in metabolic and structural integrity (Simon et al. 2022). Chronic and excessive EtOH consumption is a well‐established contributor to numerous diseases, including liver cirrhosis, cardiovascular dysfunction, myopathy, neurodegeneration, and various forms of cancer (Shield et al. 2013). These pathological outcomes reflect the systemic nature of EtOH toxicity and underscore the need for a more comprehensive understanding of its biological consequences.

A central mechanism driving EtOH‐induced tissue injury is the disruption of metabolic homeostasis. EtOH interferes with lipid metabolism at multiple levels, affecting synthesis, degradation, and transport, which leads to abnormal lipid accumulation and altered membrane composition (Bergheim et al. 2005; Steiner and Lang 2017). For example, hepatic steatosis is a hallmark of alcoholic liver disease, and EtOH‐induced changes in phospholipid profiles have been documented not only in the liver but also in the pancreas, brain, heart, and skeletal muscle (Aleynik et al. 1999; Arumugam et al. 2023; Fernando et al. 2011; Wang et al. 2019; Willis et al. 2026). These lipid disturbances contribute to cellular stress and organ dysfunction, positioning lipid dysregulation as a key mediator of EtOH‐related pathology (Lieber 2004).

Despite significant insights from clinical and preclinical studies, several limitations continue to impede a holistic understanding of EtOH's systemic effects. Many investigations have focused on individual tissues in isolation, neglecting dynamic inter‐organ interactions and broader physiological contexts. Furthermore, numerous preclinical models lack translational relevance due to non‐physiological study designs, such as unrealistic EtOH dosages, brief exposure durations, and the use of age‐inappropriate animal subjects (D'Souza El‐Guindy et al. 2010; Nieto et al. 2021; Yoladı et al. 2023). As a result, the long‐term metabolic consequences and tissue‐level disturbances induced by EtOH remain poorly characterized. These limitations constrain the applicability of current findings and underscore the need for integrative approaches that more accurately reflect the complexity of EtOH's impact across biological systems.

To address these gaps, the present study investigates the systemic effects of chronic EtOH exposure using a physiologically relevant preclinical model. This model incorporates age‐appropriate mice, realistic dosing regimens, and extended exposure durations. By simultaneously examining multiple metabolic tissues and accounting for inter‐organ interactions, we aim to provide a more integrated perspective on EtOH‐induced lipid dysregulation and its role in tissue injury. Through this approach, we seek to uncover novel insights into coordinated physiological responses to EtOH and identify mechanisms that may be obscured when tissues are studied in isolation. Ultimately, this work aims to enhance the translational relevance of preclinical EtOH research and inform future strategies for mitigating its harmful effects.

## 2 Methods and Materials

### 2.1 Ethics Approval and Animal Models

Seventeen female C57BL/6 mice were obtained from Jackson Laboratory and aged to approximately 13 weeks prior to study initiation. At the study completion, mice were anesthetized with 2%–3% isoflurane and euthanized by exsanguination in accordance with the Ohio University Animal Care and Use Committee.

### 2.2 Ethanol (EtOH) Feeding

**FIGURE 1:**
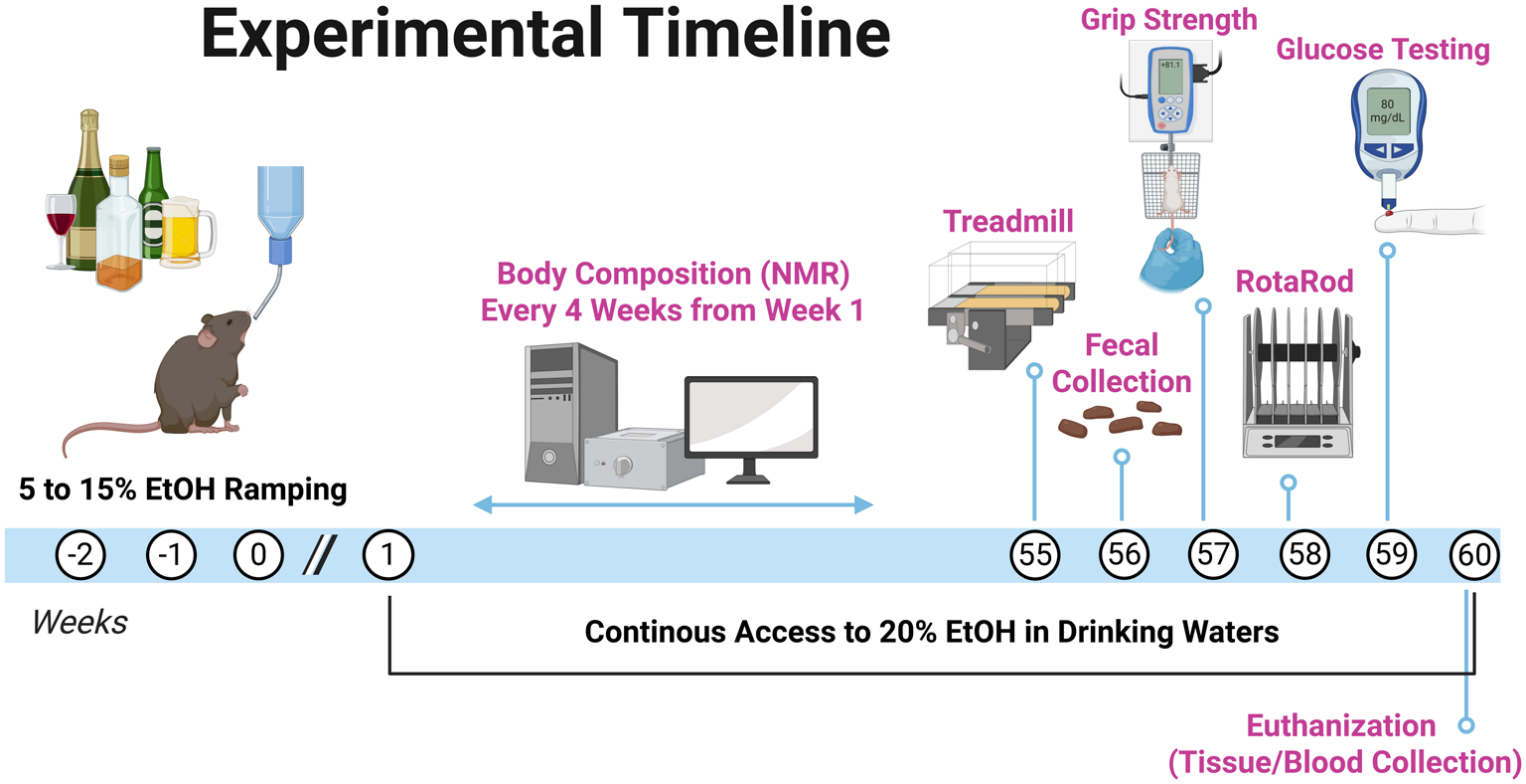
Experimental timeline. Female C57BL/6 mice began consuming 20% EtOH at 15 weeks of age. Weekly measurements included food and liquid intake and body mass; lean and fat mass were assessed every 4 weeks. By Week 52, group differences in body composition emerged, prompting additional tests (Weeks 55–59) of physical performance and glucose regulation. Fecal samples were collected at Week 56 for microbiome analysis. At ~73 weeks of age, mice were euthanized and tissues collected for analysis of lipid metabolism, membrane damage, and inflammation. Figure was created using BioRender.

Mice (*n* = 10) were randomly assigned to receive 20% EtOH in their drinking water (EtOH group) via a no‐choice design (Dekeyser et al. 2013; Moser et al. 2022; Song et al. 2002). Mice were initially acclimated to EtOH by increasing the EtOH concentration in 5% increments from 0% to the target 20% (w/v) over the course of approximately 2 weeks and then maintained at 20% until study completion (Figure 1) (Dekeyser et al. 2013; Moser et al. 2022; Song et al. 2002). The target of 20% EtOH in the drinking water was selected because it has been shown to produce similar blood alcohol concentrations (BACs) reported in chronic alcoholics (Collins and Neafsey 2016; Song et al. 2002) and be an appropriate laboratory model of alcohol‐induced organ damage. Our laboratory has previously reported BACs of ~165 mg/dL in C57BL/6 male and female mice using this EtOH feeding regimen (Moser et al. 2022, 2023). Seven mice served as controls (control group) and were given 100% water over the duration of the study. Pellet diet (ProLab 5P00), fluid, and EtOH were all consumed *ad libitum* and measured weekly. Water bottles were filled every week to ensure fluid levels remained consistent between cages.

### 2.3 Experimental Design

Although alcohol use often begins during adolescence, alcohol use disorder is more commonly diagnosed in individuals in their 20s and 30s. However, alcohol‐induced tissue damage typically manifests later in life. For example, liver conditions such as alcohol‐associated steatosis and hepatitis generally develop after decades of heavy alcohol consumption, with an average intake of approximately 120 g per day. These diseases typically present between the ages of 40 and 60. Thus, it often takes many years of sustained alcohol abuse throughout adulthood to produce measurable signs of tissue dysfunction (Lucey et al. 2009; Naveau et al. 1997). With this timeline in mind, we used an experimental protocol in mice to model systemic alcohol‐induced tissue damage in a way that is translatable to human pathology.

By 15 weeks of age, considered young adulthood, mice completed the ramping protocol and began consuming a target concentration of 20% EtOH. To assess the effects of EtOH on food and liquid intake, body mass, and body composition (i.e., lean and fat mass), a longitudinal repeated‐measures study was conducted. Food and liquid intake, along with body mass, were recorded weekly, while lean and fat mass were measured every 4 weeks (Figure 1).

By Week 52 of EtOH consumption, significant differences in body mass and composition emerged between groups. In response, we conducted additional experiments between Weeks 55 and 59 to evaluate physical performance metrics, including running endurance, grip strength, walking speed, and blood glucose regulation. During Week 56, fecal samples were collected to analyze gut microbiome composition and short‐chain fatty acid (SCFA) content. After 60 weeks of consuming 20% EtOH, mice reached 73 weeks of age and were considered aged adults. At this stage, mice were euthanized, and blood and tissue samples were collected (Figure 1). Tissues were weighed and flash‐frozen for further analysis, with particular focus on the liver and subcutaneous white adipose tissue (WAT) due to their roles in lipid regulation. Various markers and assays related to lipid metabolism, membrane damage, and inflammation were assessed in blood, liver, and WAT to characterize EtOH‐induced tissue injury. Additionally, a subset of liver tissue was prepared for histopathological analysis to evaluate evidence of pathology.

### 2.4 Experimental Procedures

#### 2.4.1 Body Composition

Mice were scanned using the Bruker Minispec nuclear magnetic resonance (NMR) Analyzer (LF50 Series, model mq 7) (Ganjayi et al. 2023). The NMR system acquires and analyzes signals from the sample volume to determine fat and lean tissue values. The system was calibrated before testing using a standard provided by the manufacturer. The mice were placed into small plastic cylinder tubes with air holes, and a tight‐fitting plunger was inserted into the cylinder to immobilize the mice. This tube was then placed into the sample chamber of the analyzer until the scan was completed, approximately 2 min.

#### 2.4.2 Running Endurance

Endurance was assessed using a time‐to‐fatigue test on a motorized treadmill (LE8700TS, Harvard Apparatus) The protocol began with a 2‐min familiarization period during which each mouse became accustomed to the treadmill environment, with the belt stationery at a 5° incline. Following the familiarization period, the exercise portion of the protocol commenced. Mice started exercising at a speed of 9 m/min, with the belt speed increasing by 3 m/min every 6 min. The treadmill incline remained at 5° incline throughout the entire protocol. Exercise motivation was provided by gently tapping the mouse's rear, as previously described (Baumann, Kwak, Ferrington, and Thompson 2018). Time to fatigue was recorded after the mouse failed to keep pace with the treadmill speed a total of three times (i.e., the mouse stayed at the back of the treadmill without attempting to re‐engage). Running endurance was defined as the total time, in seconds, that the mouse remained on the treadmill.

#### 2.4.3 Grip Strength

Grip strength was evaluated using a grip meter test (Grip meter; Model GT3, Bioseb) (Baumann, Kwak, and Thompson 2018). Mice were gently lowered over the top of a wire grid so that the front and hind paws gripped the grid (i.e., all‐limb grip strength). The tail of each mouse was pulled back steadily, keeping the mouse's torso in a horizontal position after the mouse had a firm grasp of the grip. When the mouse was unable to maintain its grip, the trial was over, and the grip strength (grams) was recorded. Each mouse performed three trials with a 10‐min rest period in‐between each trial. The best score of these trials was used as peak grip strength.

#### 2.4.4 Walking Speed

Walking speed was evaluated using a rotarod (RotaRod; LE8205, Harvard Apparatus) (Baumann, Kwak, and Thompson 2018). As a warm‐up, mice were placed on the rotarod and walked at 4 rpm for 30 s. Following the warm‐up, rotarod speed increased 1 rpm every 8 s up to 40 rpm over a 5 min period. Walking speed was recorded when the mouse was unable to sustain the rotation speed of the rotarod. Each mouse performed three trials with a 10‐min rest period in between each trial. The best score of these trials, recorded as seconds, was used as walking speed.

#### 2.4.5 Glucose Tolerance

Glucose tolerance testing was performed as previously described (Ganjayi et al. 2024), with slight modifications. Mice were fasted for 6 h before receiving an intraperitoneal (i.p.) administration of glucose (2 g/kg body weight). Blood samples were collected via tail snips immediately before (resting, or 0 min) and at 15, 30, 60, 90, and 120 min post‐glucose administration. Glucose levels were measured using a portable glucometer (TD‐4116, Metene).

#### 2.4.6 Gut Microbial Profiles

Microbiome analysis was performed by SeqCenter (Pittsburgh, PA, USA). Briefly, bacterial genomic DNA was extracted from frozen fecal samples using the ZymoBIOMICS DNA Miniprep Kit (Zymo Research). The V3‐V4 hypervariable region of the bacterial 16S rRNA gene was amplified using the universal primers 341F (5′‐CCTAYGGGNBGCWGCAG‐3′) and 806R (5′‐GACTACNVGGGTMTCTAATCC‐3′). Amplicon libraries were prepared, cleaned, normalized, and sequenced on an Illumina NextSeq2000 platform using a P1 600‐cycle flow cell to generate 2 × 301 bp paired‐end reads. Bioinformatic processing was performed using QIIME2 (v2023.9.1). Sequences were denoised (DADA2), and amplicon sequence variants (ASVs) were classified against the SILVA 138 database. The feature table was rarefied to 1000 sequences per sample for diversity analyses. Alpha diversity (Shannon, Observed ASVs, Faith's PD, Pielou's Evenness) and beta diversity (Bray‐Curtis, Jaccard, weighted/unweighted UniFrac) were calculated. Differentially abundant taxa were identified using LEfSe (Kruskal–Wallis *α* = 0.05, LDA score > 2.0). All raw sequencing data underlying the gut microbiome analyses have been deposited in an open‐access repository and are publicly available (PRJNA1426381).

#### 2.4.7 Fecal Short‐Chain Fatty Acids (SCFAs)

The SCFA extraction was followed by the protocol described by (Zhao et al. 2006). The fecal samples were resuspended in MilliQ‐grade water and homogenized using an MP Bio FastPrep system for 1 min at 4.0 m/s. The suspensions were acidified to a final pH of 2.0 using 5 M HCl. After acidification, the suspensions were incubated and centrifuged at 10,000 RPM to separate the supernatant. The resulting supernatant was spiked with 2‐ethylbutyric acid to achieve a final concentration of 1 mM. Extracted SCFA supernatants were stored in 2‐mL gas chromatography (GC) vials equipped with glass inserts. SCFA analysis was performed using a Thermo Trace 1310 gas chromatograph coupled with a flame ionization detector (FID) from Thermo Scientific. Separation was achieved using a Thermo TG‐WAXMS A GC column (30 m, 0.32 mm ID, 0.25 μm film thickness), closely following the instrumentation and methodology described in Zhao et al. (2006). The following settings were used for detection, Flame Ionization Detector Temperature: 240°C, Hydrogen: 35.0 mL/min, Air: 350.0 mL/min and makeup gas (Nitrogen) 40.0 mL/min.

#### 2.4.8 Serum Markers of Liver Damage

Blood samples were collected via cardiac puncture under anesthesia at the study's endpoint, immediately following euthanasia. Blood was allowed to clot at room temperature for 30 min, then centrifuged at 2000×*g* for 10 min at 4°C to obtain serum. Serum samples were stored at −80°C until analysis. Serum levels of alanine transaminase (ALT) (Cat # A7526‐150, Med Test) and aspartate transaminase (AST) (Cat # A7561‐150, Med Test) were measured using commercially available enzymatic colorimetric assays, following the manufacturer's instructions. Briefly, 10 μL of serum was mixed with 100 μL assay reagents in a 96‐well plate, incubated at 37°C, and absorbance was measured at 340 nm using a microplate reader (BioTek Synergy H1, Agilent). Enzyme activities were quantified against standard curves prepared with calibrators provided in the kits and expressed as units per liter (U/L). All measurements were performed in duplicate to ensure reproducibility.

#### 2.4.9 Serum Markers of Gut Permeability

Serum lipopolysaccharide (LPS) and zonulin were quantified using enzyme‐linked immunosorbent assays (ELISAs) (LPS: Cat # EKF60245, Biomatik and Zonulin: Cat # MBS2603528, Mybiosources) following the manufacturer's instructions. For LPS, 100 μL sample/standard/biotin antigen compounds were added to a 96‐well plate pre‐coated with anti‐LPS antibody and incubated for 45 min at 37°C, followed by three washes; 100 μL of LPS‐horseradish peroxidase (HRP) conjugate was added. After incubation at 37°C for 30 min, wells were washed five times, each wash lasting for 2 min, and 90 μL of tetramethylbenzidine (TMB) substrate was added for 15 min at room temperature in the dark. The reaction was stopped with 50 μL of stop solution, and absorbance was measured at 450 nm. LPS concentrations were calculated from a standard curve. For zonulin, 100 μL of diluted sample was added to a pre‐coated anti‐zonulin plate, incubated at 37°C for 90 min, washed two times with wash buffer, and incubated with 100 μL of biotinylated detection antibody for 60 min at 37°C. After three washes, 100 μL of streptavidin‐HRP conjugate was added for 30 min, followed by five washes, 100 μL of TMB for 20 min, and 100 μL of stop solution. Absorbance was read at 450 nm, and zonulin concentrations were determined from a standard curve. Both assays were performed in duplicate using a microplate reader (BioTek Synergy H1, Agilent), with blanks ensuring assay validity.

#### 2.4.10 Contents of Specific Liver Proteins

Liver tissue was homogenized in ice‐cold laboratory lysis buffer with following constituents (in mmol L^−1^): 250 sucrose, 100 KCl, 20 MOPS, and 5 EDTA (pH 6.8) supplemented with a 100× protease inhibitor cocktail (Thermo Scientific). Homogenized samples were kept as totals or centrifuged at 20,000*g* for 15 min at 4°C (i.e., supernatant). Protein contents of the total homogenate or supernatant were quantified using the A280 method on a Nanodrop spectrophotometer. Equal amounts of protein (20–30 μg) were loaded onto 10%–12% SDS polyacrylamide gels and separated according to molecular weight (100 V for 30 min followed by 150 V). Proteins were transferred to a PVDF membrane using a mini trans‐blot SD transfer system at a constant voltage of 22 V for 60 min (Bio‐Rad Laboratories) and blocked in 5% nonfat dried milk or BSA (w/v) dissolved in tris‐buffered or phosphate saline with 0.1% Tween‐20 (PBS‐T or TBS‐T) for 1 h at room temperature. Following the block, membranes were incubated with primary antibodies (Table S1) for 1 h at room temperature or overnight at 4°C with orbital rocking. Following incubation in the primary antibodies, membranes were washed with TBS‐T or PBS‐T (3 × 10 min). They were then probed with either anti‐rabbit or ‐mouse horseradish peroxidase (HRP) conjugated or Dylight secondary antibodies for 1 h at room temperature with orbital rocking and washed as previously stated. When necessary, membranes were treated with an ECL solution (Bio‐Rad Laboratories) prior to detection using a Bio‐Rad ChemiDoc XRS+ (Bio‐Rad Laboratories) and analyzed by densitometry using image lab software (Bio‐Rad Laboratories) or visualized on LI‐COR's Odyssey Infrared Imaging System and analyzed for densitometry using ImageJ (NIH, Maryland). All proteins were normalized to β‐actin unless stated otherwise.

For hepatic serum amyloid A quantification, 100 mg of liver tissue was homogenized in 300 μL of icecold‐phosphate‐buffered saline using a bead‐based homogenizer on ice. Homogenates were centrifuged, and the resulting supernatant was collected and either analyzed immediately or stored at −80°C. serum amyloid A concentrations were measured using the Quantikine Mouse Serum Amyloid A Immunoassay (R&D Systems; MSAA00) according to the manufacturer's instructions with modifications for tissue samples; liver supernatants were diluted 1:10 in Calibrator Diluent RD5‐26. Samples, standards, and controls (50 μL/well) were added in duplicate to antibody‐coated 96‐well plates and incubated for 2 h at room temperature with orbital shaking. Plates were washed, then incubated for an additional 2 h with HRP‐conjugated anti‐mouse serum amyloid A detection antibody. After washing, TMB substrate was added and allowed to develop for 30 min at room temperature protected from light. Reactions were stopped with 2N sulfuric acid, and absorbance was measured at 450 nm with 540 nm correction. All samples were run in duplicate, and serum amyloid A concentrations were calculated from the standard curve.

#### 2.4.11 Serum Lipid Profiles

Serum lipid profiles including free fatty acids (FFAs), triglycerides, and total cholesterol were measured using enzymatic colorimetric assay kits (FFA: Cat # 102965‐238, Cell Biolabs; triglycerides: Cat # MA‐TG, Ray Bio Laboratories; and total cholesterol: Cat # EHDL004, BioAssay Systems) per the manufacturers' protocols. For FFAs, 10 μL of serum was added to a 96‐well plate with 200 μL of 1× enzyme mixture and incubated at 37°C for 30 min; 100 μL of the above detection enzyme mixture was then added to each well, incubated for 10 min at 37°C and absorbance was measured at 540 nm. Concentrations were calculated from a standard curve. For triglycerides, 10 μL of diluted serum in assay buffer was added with 100 μL of reaction mix containing lipase, glycerol kinase, glycerol phosphate oxidase, and incubated at 37°C for 10 min; absorbance was read at 570 nm, with concentrations determined from a glycerol standard curve. For total cholesterol, 50 μL of diluted serum was added to 150 μL of reaction mix containing cholesterol esterase, cholesterol oxidase, HRP, and chromogen, incubated at 37°C for 30 min; absorbance was measured at 570 nm, with concentrations calculated from a standard curve. All assays were performed in duplicate using a microplate reader (BioTek Synergy H1, Agilent), with kit‐provided blanks used for background correction.

#### 2.4.12 Liver Lipid Profiles

Extraction and subsequent quantification of hepatic triglycerides and total cholesterol were conducted using enzymatic assays as previously described (Helsley et al. 2025; Zelows et al. 2023). In brief, initial liver mass was recorded then samples were delipidated in 4 mL of 2:1 chloroform to methanol (v/v) overnight. A dilute concentration of H2SO4 (0.05%) was added (1.2 mL), samples were vortexed then centrifuged at 2000 rpm (15 min) for phase separation. The bottom (organic) phase was recorded, and 0.5 mL of the organic phase was added to 1 mL of 1% TritonX‐100 in chloroform. The samples were then dried under N_2_ and 0.5 mL H_2_O was added prior to quantification of triglycerides (Wako), total and free cholesterol (Wako) per manufacturer's instructions. All standards and blanks were prepared in a similar fashion, and data were expressed as μg lipid/mg of wet tissue.

#### 2.4.13 Liver Pathophysiology

Liver tissues were fixed in 10% neutral‐buffered formalin for 24–48 h, processed through graded ethanol and xylene, embedded in paraffin, and sectioned at 5 μm. Sections were stained with hematoxylin and eosin (H&E) using Harris hematoxylin for 5 min, followed by differentiation in acid alcohol, bluing in tap water, and counterstaining with 0.5% eosin Y for 1 min. Slides were then dehydrated, cleared in xylene, mounted, and examined by light microscopy.

#### 2.4.14 Liver Amyloid‐β Content

Mouse amyloid‐β42 levels were quantified using an ELISA kit (Invitrogen KMB3441) according to the manufacturer's instructions. Liver tissue (~50 mg) was homogenized in 400 μL of 5 M guanidine‐HCl buffer (50 mM Tris–HCl, pH 8.0) with protease inhibitors, incubated at room temperature for 3 h, diluted 1:10 in PBS, and centrifuged at 16,000×*g* for 20 min at 4°C. Supernatants were collected and stored at −80°C. The lyophilized standard was reconstituted in carbonate–bicarbonate buffer (pH 9.0) and serially diluted to generate a standard curve (200, 100, 50, 25, 12.5, 6.25, and 3.12 pg/mL). All standards and samples were assayed in duplicate. Briefly, 100 μL of each standard, blank, or sample was added to pre‐coated wells and incubated for 2 h at room temperature. After washing, 100 μL of biotinylated detection antibody was added for 1 h, followed by streptavidin‐HRP for 30 min at RT. After final washes, 100 μL of stabilized chromogen (TMB) substrate was added for 30 min in the dark, the reaction was stopped with 2 N H₂SO₄, and absorbance was read at 450 nm. Mouse Aβ42 concentrations were calculated from the standard curve.

#### 2.4.15 Tissue Free Fatty Acids

Free fatty acids (FFAs) in subcutaneous WAT were quantified using an enzymatic colorimetric assay kit (Cat # E‐BC‐K013‐S, Elabscience Laboratories). WAT (100 mg) was homogenized in extracting solution and centrifuged at 10000*g* for 10 min at 4°C, assayed by adding 1 mL to tube with 500 μL enzyme reaction mix, oscillated for 5 min, incubated at room temperature for 5 min, and measured for OD at 715 nm using a spectrophotometer. FFA concentrations (μmol/g tissue) were calculated from a standard curve in duplicate.

#### 2.4.16 Transcriptomics

RNA was isolated from WAT using a Takara NucleoSpin RNA kit according to the manufacturer's instructions, and 300 ng total RNA was sequenced using 100 base pair paired end reads at a total depth of approximately 40 million reads per sample. Genomic mapping of sequence reads, and differential expression analysis was done in RStudio v2025.09.1 with paired end read mapping done via Rsubread v2.18.0 (Liao et al. 2019). Mapped genes were filtered to only those that mapped in at least six samples, and differential expression analysis was subsequently performed using DESeq2 v1.44.0 (Love et al. 2014), using an exploratory threshold of a false discovery rate (FDR)‐corrected *p*‐value ≤ 0.1. Gene Ontology analysis of differentially expressed downregulated genes was done using GO. db v3.19.1 and AnnotationDbi v3.19 (Pagès Hervé 2025), applying a statistical threshold of FDR *p*‐value ≤ 0.01 and ≥ 3 total genes per GO term. All RNA‐seq raw data files and total gene count files are deposited on the NCBI GenBank (GSE324693).

### 2.5 Statistical Analyses

Changes across time and group were assessed using a two‐way ANOVA. A Sidak post hoc test was performed in the event of a significant ANOVA. To determine differences between the EtOH and control groups, an unpaired *t*‐test was utilized, with Welch's correction applied when standard deviations were unequal between groups. A *p*‐value of < 0.05 was required for statistical significance. A Bonferroni correction was applied when assessing group differences for bacterial genera (*p* < 0.007) and SCFAs (*p* < 0.006). Values are presented as mean ± error of the mean (SEM). All statistical testing was performed using GraphPad Prism 10.6.0 (GraphPad Software).

## 3 Results

### 3.1 EtOH Disrupts Energy Balance, Body Composition and Physical Function

**FIGURE 2:**
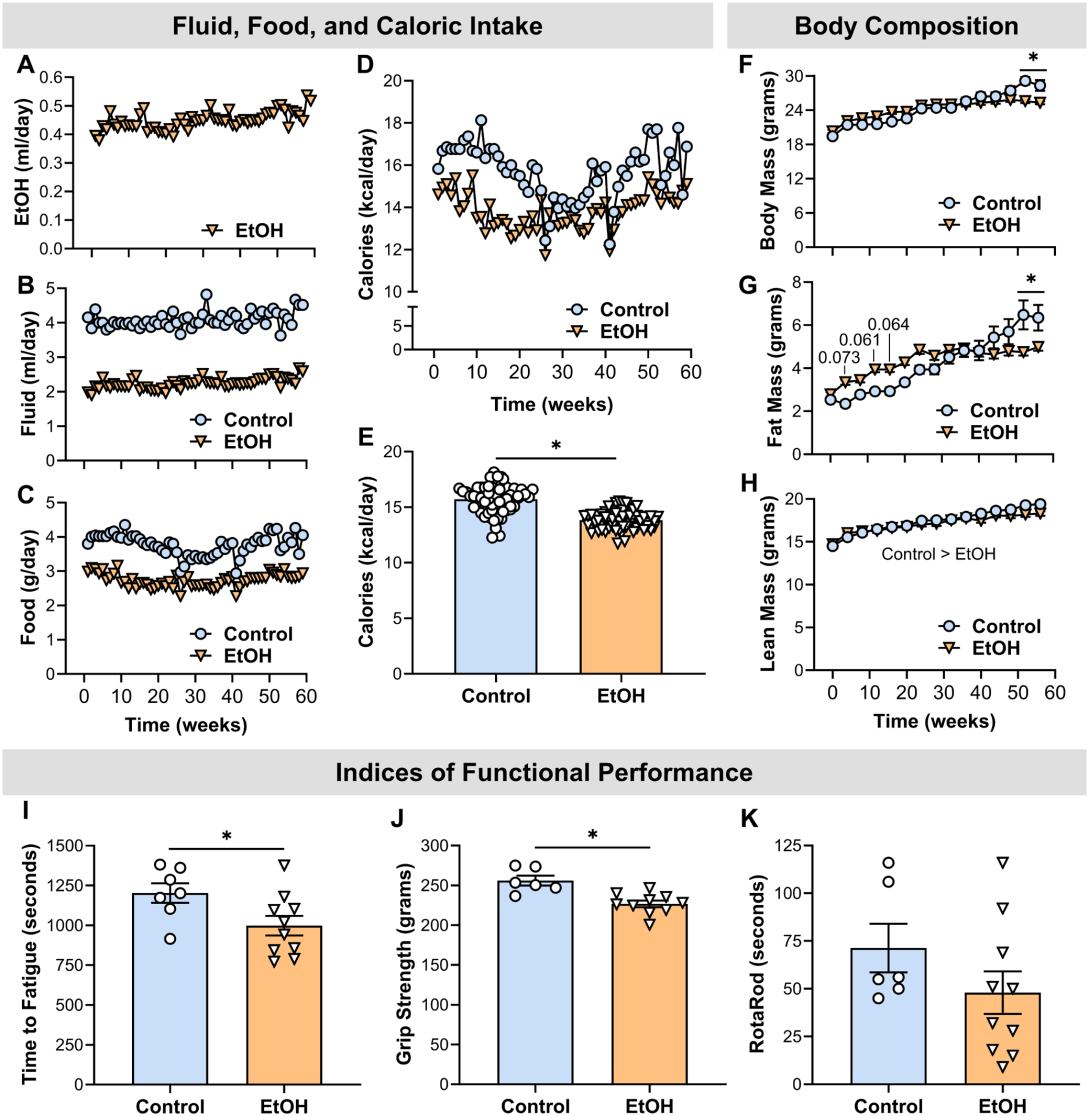
Chronic EtOH consumption disrupts energy balance, body composition, and physical function. (A) Daily voluntary EtOH intake (mL) over the 60‐week experimental period. (B–D) Average daily total fluid intake, chow diet intake, and total caloric intake in control and EtOH‐fed mice. (E) Longitudinal profile of total caloric intake for 60 weeks. (F–H) Body mass, fat mass, and lean mass measured every 4 weeks. (I–K) Physical performance assessments: Treadmill endurance (Week 55), forelimb grip strength (Week 57), and RotaRod performance (Week 58). Statistical analyses included unpaired *t*‐tests, Welch's *t*‐tests, or two‐way ANOVAs. *Significantly different from control group (*p* < 0.05). Data are presented as mean ± SEM. Samples sizes were 6–7 for controls and 9–10 for EtOH.

Mice assigned to the EtOH cohort maintained a consistent voluntary intake of 0.4–0.5 mL of EtOH per day over the 60‐week experimental period (Figure 2A). This level of EtOH consumption was associated with a > 30% reduction in both chow and total fluid intake relative to control animals (Figure 2B,C). When EtOH‐derived calories were included in the total energy intake, the EtOH group exhibited a modest but statistically significant 12% reduction in average daily caloric intake compared to controls (*p* < 0.001) (Figure 2E). This caloric deficit was more pronounced during the initial 30 weeks of exposure and attenuated during the latter half of the study (Figure 2D).

Despite sustained reductions in fluid and caloric intake, body mass remained comparable between groups until week 52, at which point the EtOH group demonstrated a significant decline (*p* < 0.001) (Figure 2F). Adiposity, measured as body fat mass, was initially elevated in the EtOH group during the first 16 weeks but subsequently declined below control levels by Week 52 (*p* ≤ 0.004) (Figure 2G). Lean mass analysis revealed a significant main effect of treatment, indicating reduced lean tissue in the EtOH group (*p* = 0.001); however, the lack of a significant time × treatment interaction precluded determination of the precise onset of this difference (*p* = 0.213) (Figure 2H).

Functional performance assessments conducted during the terminal phase (Weeks 55–59) revealed significant impairments in the EtOH group. Specifically, treadmill endurance and grip strength were reduced by 17% and 11%, respectively, compared to controls (*p* ≤ 0.037) (Figure 2I,J). No significant differences were observed in motor coordination, as assessed by RotaRod performance (*p* = 0.203) (Figure 2K).

### 3.2 EtOH Drives Taxonomic Shifts in Gut Microbiota and Depletes Key SCFAs

**FIGURE 3:**
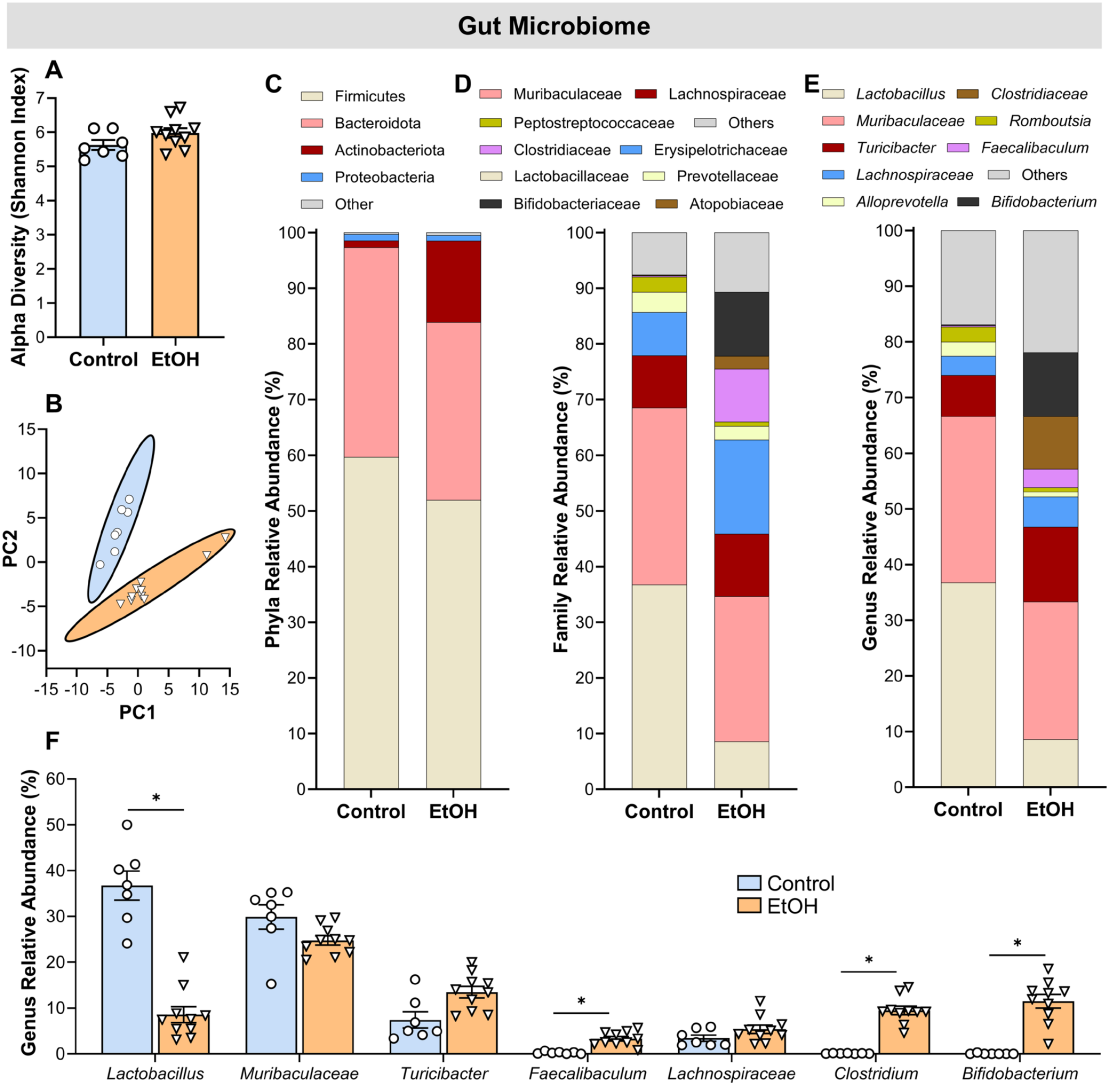
Chronic EtOH exposure alters gut microbiota composition. (A) Alpha diversity of the gut microbiome (Shannon Index). (B) Principal Component Analysis (PCA) of microbial communities. (C–E) Relative abundances of major microbial taxa at the phylum, family, and genus levels. (F) Significantly altered bacterial genera between control and EtOH groups. Gut microbiota was analyzed from fecal samples collected at week 56 using 16S rRNA sequencing. Statistical analyses included unpaired t‐tests or Welch's *t*‐tests. *Significantly different from control group was set to *p* < 0.007 because a Bonferroni correction was applied when assessing group differences for each bacterial genera. For alpha diversity, *p* < 0.05. Data are presented as mean ± SEM. Samples sizes were 7 for controls and 10 for EtOH.

Chronic EtOH exposure did not significantly affect gut microbiome alpha diversity (*p* = 0.104) (Figure 3A), but it induced substantial shifts in the relative abundance of specific microbial taxa as shown by Principal Component Analyses (Figure 3B). Distinct compositional differences were observed between EtOH and control groups at the phylum, family, and genus levels (Figure 3C–E). At the genus level (using a Bonferroni corrected *p*‐value of < 0.007), EtOH consumption was associated with a marked reduction in *Lactobacillus* (*p* < 0.001) while *Faecalibaculum*, *Clostridium*, and *Bifidobacterium* were significantly enriched (*p* < 0.001) (Figure 3F). No group differences were observed in *Muribaculaceae*, *Turicibacter*, or *Lachnospiraceae* (*p* ≥ 0.012) (Figure 3F).

**FIGURE 4:**
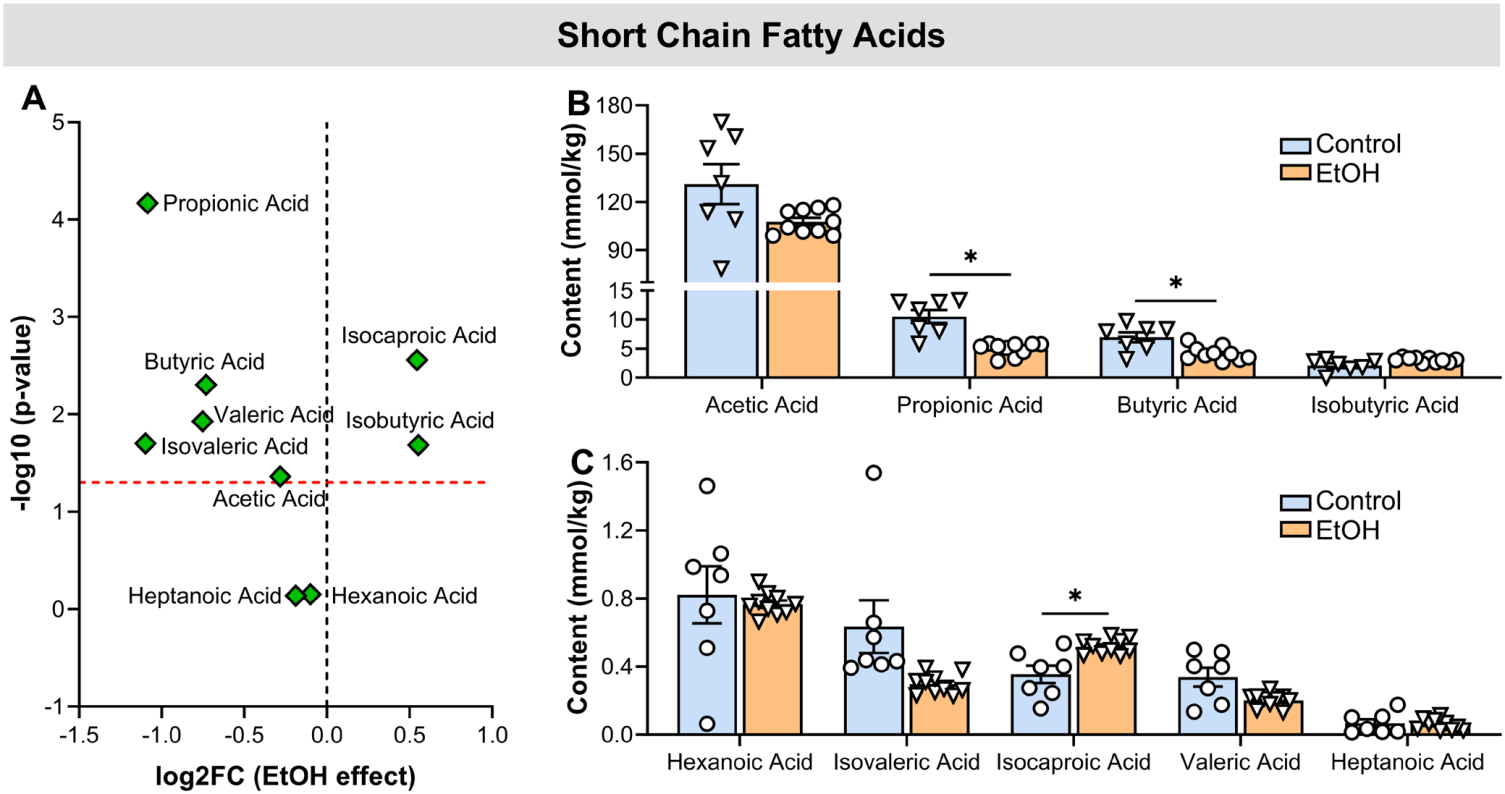
Chronic EtOH consumption reduces fecal SCFAs. (A) *Z*‐scores for all measured SCFAs. (B, C) Fecal concentrations of SCFAs. SCFA levels were measured in fecal samples collected at week 56 using gas chromatography–mass spectrometry (GC–MS). Statistical analyses included unpaired t‐tests or Welch's t‐tests. *Significantly different from control group was set to *p* < 0.006 because a Bonferroni correction was applied when assessing group differences for each SCFA. Data are presented as mean ± SEM. Samples sizes were 7 for controls and 10 for EtOH.

In parallel, fecal concentrations of the primary SCFAs (using a Bonferroni corrected *p*‐value of < 0.006), propionic and butyric acids were reduced (*p* ≤ 0.005) and isocaproic acid was increased (*p* = 0.003) following chronic EtOH consumption (Figure 4A–C). Levels of isovaleric, valeric, isobutyric, and hexanoic acids (*p* ≥ 0.012) and the medium‐chain fatty acid heptanoic acid (*p* = 0.733) were found to be similar between the EtOH and control groups (Figure 4A,C).

### 3.3 EtOH Promotes “Leaky Gut”, Induces Liver Inflammation & Amyloidosis and Impairs Cholesterol Biosynthesis

**FIGURE 5:**
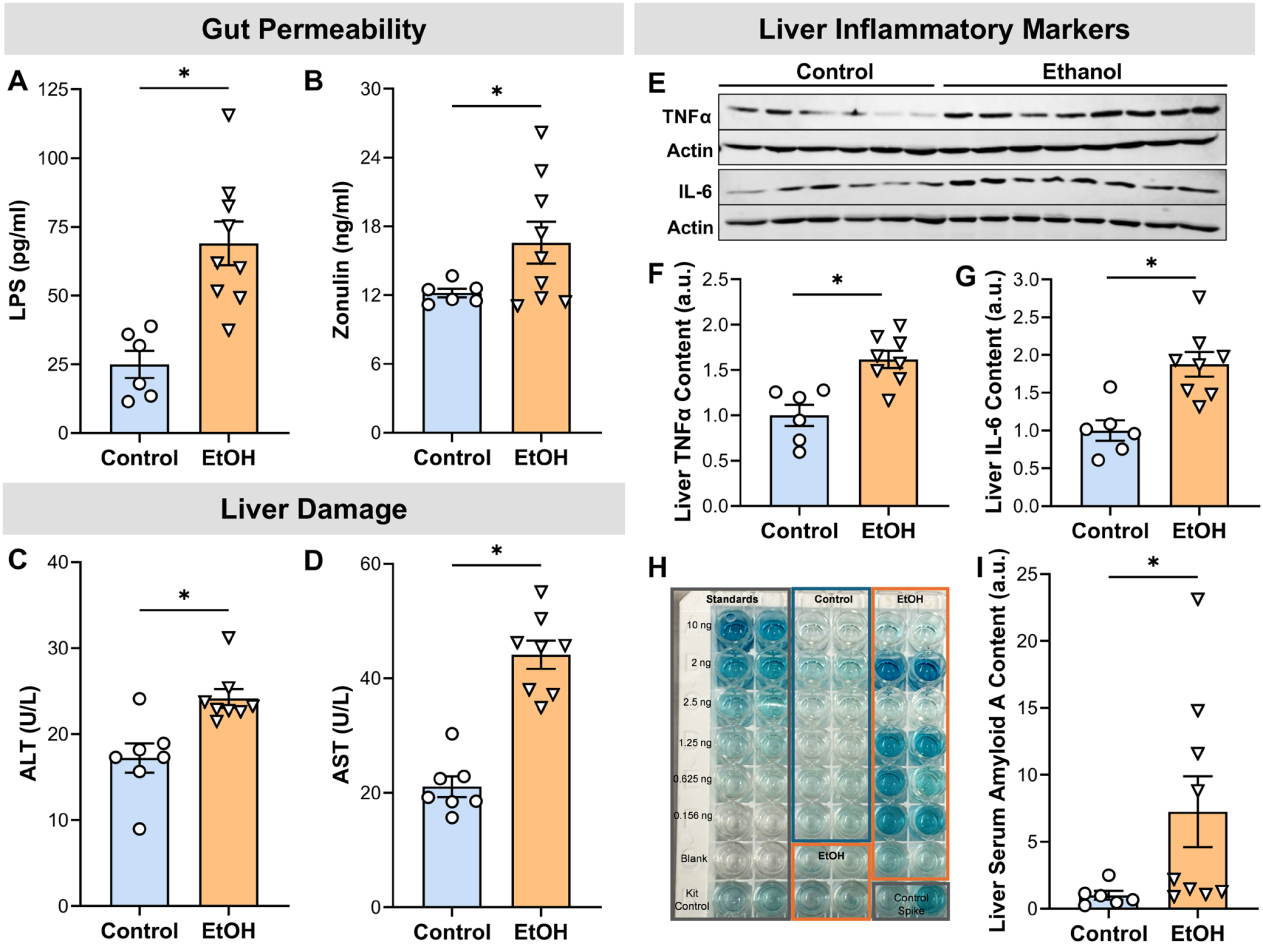
Chronic EtOH induces gut permeability, hepatic injury & inflammation. (A, B) Indirect makers of intestinal barrier dysfunction: Serum LPS and zonulin. (C, D) Serum markers of hepatic injury: ALT and AST. (E–I) Liver pro‐inflammatory markers: TNFα and IL‐6 protein expression, and the acute‐phase protein serum amyloid A quantified using a colorimetric ELISA (Panel H; gray outlines indicate standards; blue, control; orange, EtOH). Statistical analyses included unpaired *t*‐tests or Welch's *t*‐tests. *Significantly different from control group (*p* < 0.05). Data are presented as mean ± SEM. Samples sizes were 6–7 for controls and 8–9 for EtOH.

Mice consuming EtOH exhibited marked disruption of intestinal barrier function, evidenced by significantly elevated serum LPS and zonulin concentrations compared with controls (*p* ≤ 0.044) (Figure 5A,B). These indirect markers of increased gut permeability were positively associated with hepatic injury, as reflected by higher serum ALT and AST levels in the EtOH group (*p* ≤ 0.004) (Figure 5C,D). In addition, EtOH exposure promoted a pro‐inflammatory hepatic environment, demonstrated by increased tumor necrosis factor‐α (TNFα) and interleukin‐6 (IL‐6) protein expression (*p* ≤ 0.002) (Figure 5E–G). Because these cytokines drive the hepatocyte acute‐phase response, hepatic serum amyloid A content was assessed and found to be elevated approximately seven‐fold relative to controls (*p* = 0.046), with substantial variability within the EtOH group yet still significantly higher than in controls (Figure 5H,I).

**FIGURE 6:**
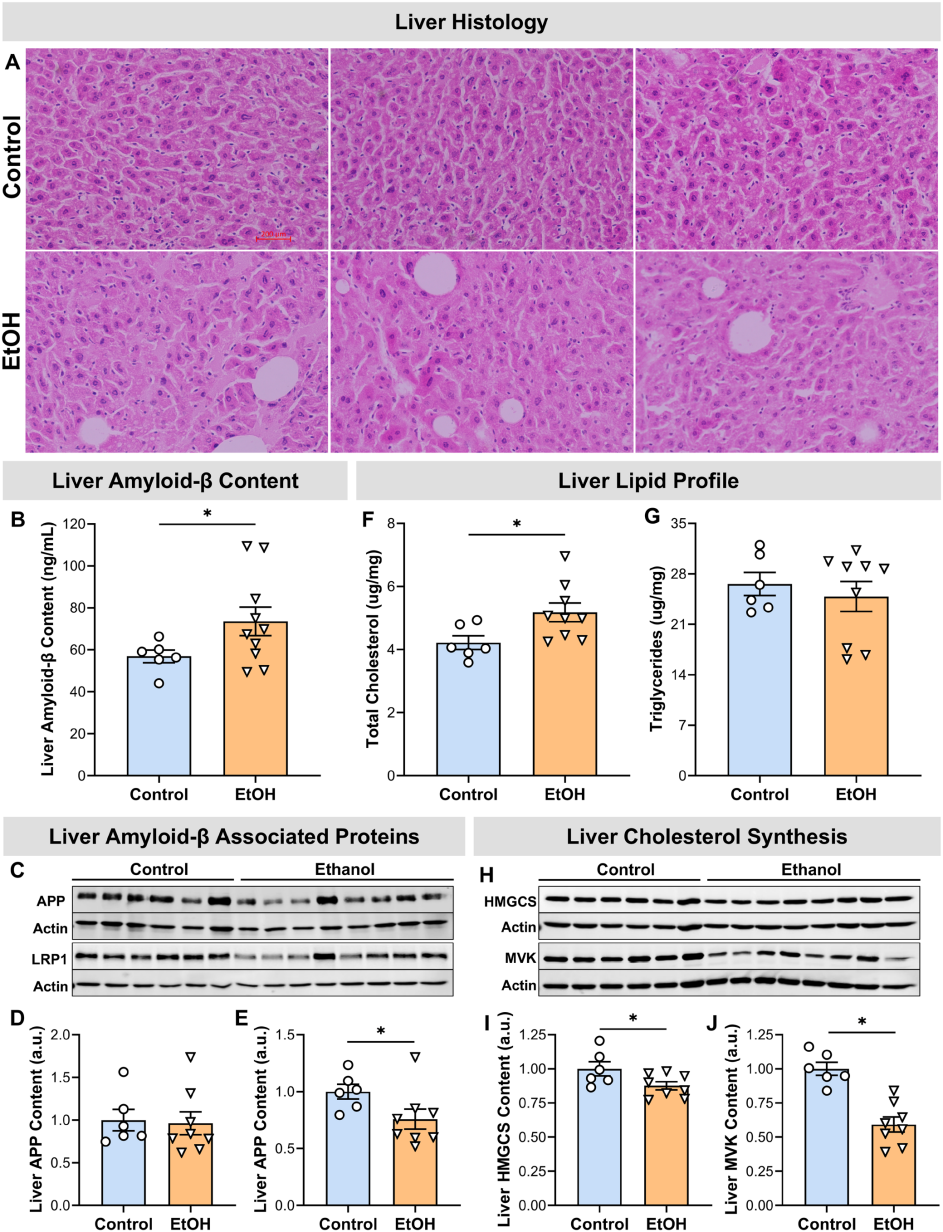
Chronic EtOH exposure induces hepatic amyloid and cholesterol accumulation. (A) Liver histology assessed by H&E staining. (B) Hepatic amyloid‐β content. (C‐E) Hepatic proteins associated with liver amyloid‐β content. (F–G) Hepatic lipid content: Total cholesterol and triglycerides. (H‐J) Liver cholesterol synthesis markers: HMGCS and MVK protein expression. Statistical analyses included unpaired t‐tests or Welch's *t*‐tests. *Significantly different from control group (*p* < 0.05). Samples sizes were 6 for controls and 8–10 for EtOH except for histology, which was 3/group.

Beyond its role in inflammatory signaling, cytokine production, and immune cell recruitment, serum amyloid A is also the precursor protein for AA amyloid, the fibril type that characterizes AA amyloidosis. Persistent elevation of serum amyloid A can lead to its misfolding and extracellular deposition as AA amyloid fibrils in the liver (Husby et al. 1988; Simons et al. 2013; Yuan et al. 2019). Accordingly, H&E staining was performed, revealing histological features consistent with hepatic amyloidosis, including extracellular deposits of acellular, homogeneous, eosinophilic (pink) hyaline material with a smooth, glassy appearance and absence of cellular or fibrillar detail (Figure 6A).

In contrast to AA amyloid, hepatic amyloid‐β derives from cleavage of the amyloid precursor protein (APP) and is mechanistically distinct. Alcohol consumption has been reported to modulate hepatic amyloid‐β biology through effects on APP and lipoprotein receptor–related protein 1 (LRP1) expression (Garcia et al. 2022). Consistent with these observations, hepatic amyloid‐β content increased by 29% in the EtOH group compared with controls (*p* = 0.047) (Figure 6B). APP levels did not differ between groups (*p* = 0.850), whereas LRP1 levels were reduced by 24% in the EtOH group relative to controls (*p* = 0.047) (Figure 6C–E).

In addition to elevated serum liver enzymes and hepatic inflammation & amyloidosis, EtOH consumption altered hepatic lipid metabolism. Total cholesterol was increased by 23% in the EtOH group (*p* = 0.035) (Figure 6F), while triglyceride levels remained unchanged (*p* = 0.558) (Figure 6G), suggesting a selective disruption of cholesterol homeostasis. Protein expression of key enzymes involved in cholesterol biosynthesis, 3‐hydroxy‐3‐methylglutaryl‐CoA synthase (HMGCS) and mevalonate kinase (MVK), was downregulated in EtOH‐treated mice (*p* ≤ 0.048) (Figure 6H–J), suggestive of cholesterol‐mediated repression of SREBP2 processing with EtOH‐feeding.

### 3.4 EtOH Causes Dyslipidemia and Glucose Intolerance With Minimal Impact on White Adipose Tissue Gene Expression

**FIGURE 7:**
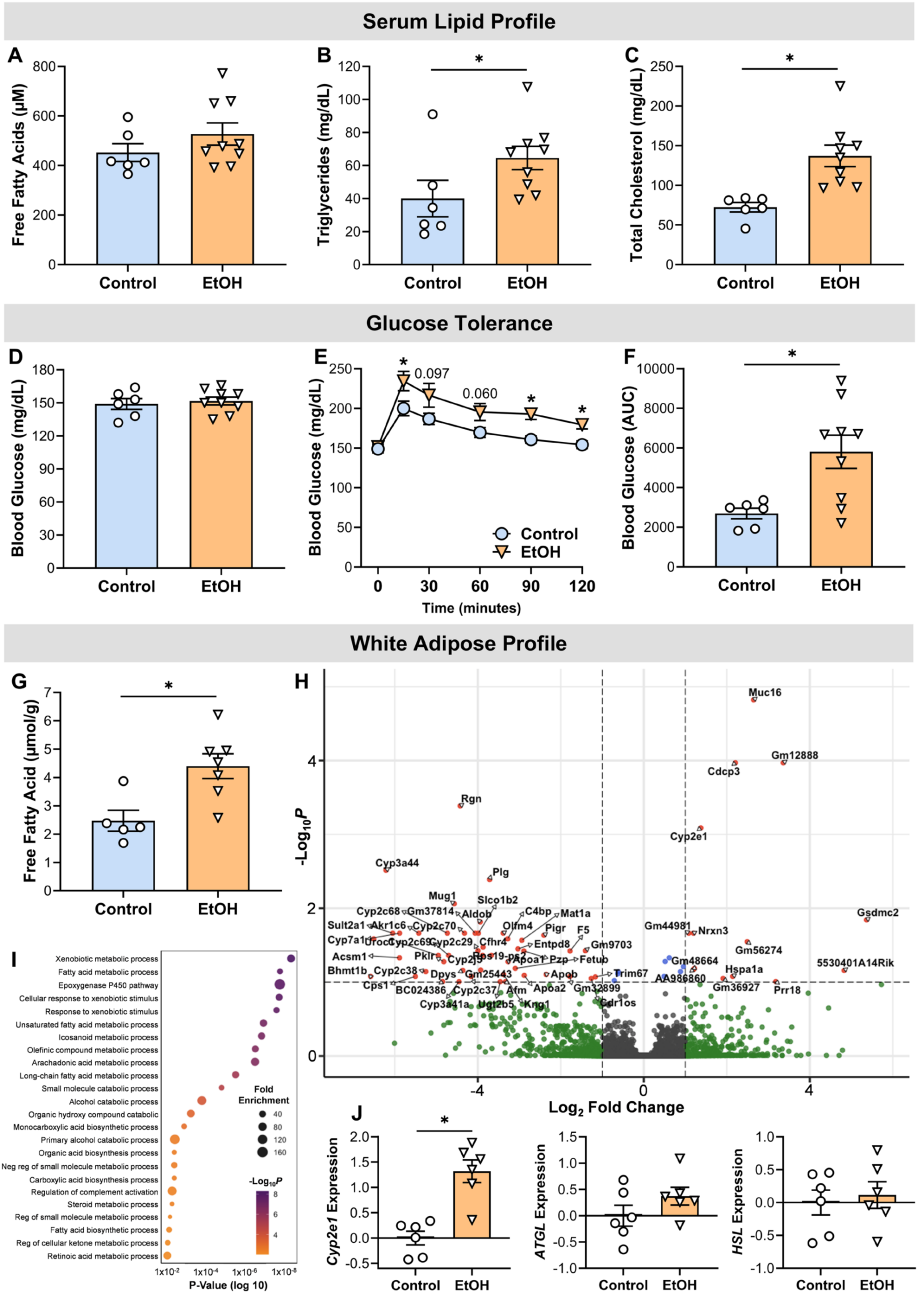
Chronic EtOH causes dyslipidemia and glucose intolerance. (A–C) Serum lipid profile: Free fatty acids, triglycerides, and total cholesterol. (D‐F) Fasting blood glucose, intraperitoneal glucose tolerance test, and area under the curve (AUC). (G) Free fatty acid content in WAT. (H) Volcano plot of differentially expressed genes in WAT: Significantly upregulated and downregulated genes are highlighted. (I) Gene Ontology (GO) enrichment analysis of biological processes for downregulated genes. (J) Subset of selectively targeted genes in WAT. Statistical analyses included unpaired *t*‐tests or Welch's *t*‐tests. *Significantly different from control group (*p* < 0.05). Data are presented as mean ± SEM. Samples sizes were 5–6 for controls and 6–9 for EtOH.

Given the observed alterations in hepatic cholesterol levels, we next evaluated circulating blood lipids. Chronic EtOH consumption did not significantly alter serum FFA concentrations (*p* = 0.215) (Figure 7A). However, EtOH‐treated mice exhibited elevated serum triglyceride and total cholesterol levels compared to controls (*p* ≤ 0.049) (Figure 7B,C), indicative of dyslipidemia.

This dyslipidemic profile was accompanied by impaired glucose tolerance, despite no significant differences in baseline fasting blood glucose (*p* = 0.660) (Figure 7D). Following administration of a glucose bolus, EtOH‐treated mice displayed significantly higher blood glucose concentrations at 15‐, 90‐, and 120‐min post‐injection relative to controls (*p* ≤ 0.041) (Figure 7E). This impairment in glucose clearance was further substantiated by an increased area under the curve (AUC) (*p* = 0.012) (Figure 7F).

Because WAT is a key regulator of systemic lipid and glucose metabolism, we assessed subcutaneous WAT FFA content and transcriptomic profiles. In contrast to serum findings, chronic EtOH consumption increased WAT FFA levels by 78% compared to controls (*p* = 0.010) (Figure 7G), suggesting local metabolic dysregulation. Despite this, only a limited number of genes were differentially expressed in WAT following EtOH exposure. Specifically, 61 genes were significantly altered, with the majority (47 genes) being downregulated (Figure 7H). Gene Ontology (GO) enrichment analysis of these downregulated genes revealed significant overrepresentation of pathways related to xenobiotic metabolism, fatty acid metabolism, and alcohol catabolism (Figure 7I). Due to the small number of upregulated genes, enrichment analysis for upregulated pathways was not considered robust. Using a targeted approach to examine *Cyp2e1, ATGL*, *HSL* (Figure 7J), genes or protein previously reported to be affected by EtOH in adipose tissue (Chen et al. 2009; Crowell et al. 2016; Lee and Lee 2015; Sebastian et al. 2011; Sun et al. 2012; Zhong et al. 2012), only *Cyp2e1* showed a significant change in gene expression following chronic EtOH consumption (*p* < 0.001).

## 4 Discussion

Chronic EtOH consumption led to widespread physiological and metabolic disturbances in mice, even in the context of modest reductions in total caloric intake. Although body mass remained stable for much of the study, prolonged EtOH exposure ultimately resulted in differences in lean mass and adiposity compared to control mice, which was accompanied by impairments in grip strength and endurance. These systemic effects coincided with marked alterations in gut microbiota composition and depletion of major SCFAs, indicating disruption of host–microbe interactions. Furthermore, EtOH intake compromised intestinal barrier integrity (as indirectly assessed via elevated circulating LPS & zonulin) and induced hepatic injury characterized by liver damage (i., increased serum ALT & AST and amyloidosis), dysregulated cholesterol biosynthesis, and pro‐inflammatory signaling. Metabolic consequences extended beyond the liver, as evidenced by dyslipidemia and impaired glucose tolerance, despite minimal transcriptional remodeling within subcutaneous WAT. Together, these findings demonstrate that chronic EtOH exposure exerts multifaceted effects on energy balance, the gut–liver axis, and global metabolic homeostasis.

Chronic alcohol consumption is a well‐established disruptor of gut homeostasis, it promotes potentially pathogenic bacteria, increases endotoxin‐mediated intestinal permeability, and depletes SCFA–producing taxa (Bjørkhaug et al. 2019; de Timary et al. 2015; Patel et al. 2015). The microbial shifts identified here fit within the complex, context‐dependent landscape of EtOH‐associated dysbiosis. A consistent feature across EtOH‐related studies is depletion of *Lactobacillus* (Bull‐Otterson et al. 2013; Engen et al. 2015), replicated in the present dataset. *Lactobacillus* species support gut barrier integrity and produce anti‐inflammatory metabolites; their reduction is frequently associated with increased intestinal permeability (Dempsey and Corr 2022; Qin et al. 2022). Broader ecological changes are less uniform. Notably, a significant enrichment of *Bifidobacterium*, a genus typically considered beneficial, was observed, consistent with previous studies of chronic EtOH consumption (Yang et al. 2024). Additional expansions included *Turicibacter*, implicated in bile acid metabolism (Lynch et al. 2023), *Faecalibaculum*, frequently enriched in murine models of metabolic dysfunction (Han et al. 2024; Martínez et al. 2013) and *Clostridium sensu stricto 1*, previously reported to increase in individuals with alcohol use disorder (Mutlu et al. 2012). Although the specific composition of EtOH‐associated dysbiosis varies among studies, the overall pattern observed here aligns with dysbiotic signatures reported in human alcoholic liver disease (Bajaj 2019), underscoring the translational relevance of this model and providing a mechanistic basis for subsequent SCFA depletion and gut–liver axis disruption.

As mentioned, the depletion of fecal SCFAs represents a critical functional disruption of the gut–liver axis. Loss of SCFA‐producing taxa, including *Lactobacillus*, impairs colonic fermentation and reduces the generation of acetate, propionate, and butyrate, consistent with both current and prior reports (Cani and Delzenne 2009; Koh et al. 2016). This pattern aligns with accumulating evidence that chronic EtOH consumption diminishes SCFA availability (Leclercq et al. 2014). Although an enrichment of *Bifidobacterium* was observed, this shift appears insufficient to compensate for the broader loss of fermentative capacity. Importantly, SCFA depletion, particularly of butyrate, is mechanistically relevant, as these metabolites are essential for maintaining epithelial barrier integrity and limiting mucosal inflammation (Parada Venegas et al. 2019). In accordance with this framework and previous studies, reduced SCFA levels in the present study were accompanied by indirect markers of increased intestinal permeability, including elevated serum LPS and zonulin (Fasano 2012; Jung et al. 2021; Szabo and Bala 2010), as well as indices of hepatic inflammation (Donepudi et al. 2018). Notably, decreased *Lactobacillus* abundance was associated with lower butyrate levels and increased endotoxemia, liver injury, and hepatic inflammatory markers, confirming a link between EtOH‐driven microbial dysbiosis and hepatic pathology. Collectively, these findings support a model in which chronic EtOH intake, even in the context of caloric restriction, induces a dysbiotic state that compromises SCFA production, weakens gut barrier integrity, and promotes microbial translocation into the portal circulation, thereby propagating gut–liver axis inflammation and exacerbating metabolic and hepatic dysfunction.

The chronic and excessive EtOH exposure used in this study likely impaired liver function through multiple mechanisms. EtOH metabolism in hepatocytes generates reactive oxygen species (ROS) and disrupts lipid homeostasis, leading to the accumulation of cholesterol, bile acids, and FFAs in the liver (Osna et al. 2017; You and Arteel 2019). Additionally, increased gut permeability elevates circulating LPS levels, which activate Kupffer cells and amplify cytokine production, thereby promoting inflammation (Osna et al. 2017; Rao et al. 2004). In our model, consumption of 20% EtOH resulted in significant liver injury, as evidenced by elevated serum ALT and AST levels and increased hepatic expression of the proinflammatory cytokines TNFα and IL‐6, and the acute‐phase protein serum amyloid A. Notably, the presence of hepatic amyloid deposition was unexpected, though not without precedent, at least in the case of amyloid‐β. It was recently found that intragastric EtOH feeding increased hepatic amyloid‐β content 61% in mice, accompanied by alterations in LRP1 and APP expression (Chandrashekar et al. 2024; Garcia et al. 2022), findings that largely align with those observed here. Despite downregulation of genes and proteins involved in cholesterol biosynthesis, we and others have reported hepatic accumulation of total cholesterol and primary bile acids following chronic EtOH exposure, including cholic acid, 7‐keto‐deoxycholic acid, and taurine‐conjugated cholic acid (Donepudi et al. 2018; Willis et al. 2026). Furthermore, levels of secondary bile acids appear reduced in the livers of EtOH‐fed mice (Willis et al. 2026), implicating disrupted gut–liver signaling in the loss of bile acid homeostasis (Brandl et al. 2018; Jew and Hsu 2023). Taken together, these observations underscore the complexity of EtOH‐induced liver injury and highlight impaired gut–liver signaling and hepatic homeostatic dysfunction as key contributors to disease progression.

EtOH‐induced disruption of gut and liver function produced systemic consequences, including dyslipidemia, glucose intolerance, and reduced physical performance. Notably, these hallmarks of alcohol exposure emerged despite modest caloric restriction (~12%), a regimen typically linked to broad health benefits (Di Francesco et al. 2018, 2024; Mercken et al. 2012). Caloric restriction is itself a potent, independent modulator of the gut ecosystem, often increasing the abundance of putatively beneficial taxa such as *Lactobacillus* and *Bifidobacterium* and enhancing SCFA production (Fontana and Partridge 2015; von Schwartzenberg et al. 2021). Accordingly, the final microbial signature, reduced *Lactobacillus* and SCFAs alongside enriched *Bifidobacterium*, likely reflects the net effect of two opposing forces: the dysbiotic pressure of chronic EtOH exposure and the restorative, probiotic influence of caloric restriction. This interaction suggests that the standalone dysbiotic and functional impact of EtOH would be even more pronounced in the absence of an energy deficit; stated differently, some detrimental effects of EtOH in the present study may be underestimated if caloric restriction conferred partial protection against EtOH‐associated tissue damage and injury. As such, the lack of detectable fibrosis or steatosis in the EtOH group may reflect the effects of calorie restriction, which has been reported to attenuate age‐related liver pathology in mice (Horrillo et al. 2013). Our previous work in High Drinking in the Dark (HDID) mice (Ganjayi et al. 2023) support that calorie restriction may have some protective effects against chronic EtOH consumption, at least the context of skeletal muscle tissue and lifespan. Indeed, HDID mice consumed more total calories than controls, despite EtOH, intake and developed myopathy earlier and had higher mortality rates compared to EtOH‐treated C57BL/6 mice (i.e., mice that were “calorie restricted”).

Although EtOH‐fed mice consumed fewer total calories, especially during the initial ~30 weeks, their body mass gain matched that of control mice through Week 52, after which the groups diverged. Consistent with prior reports (Crowell et al. 2016; Moser et al. 2023), EtOH‐exposed mice ultimately exhibited lower body mass than controls, largely because controls progressively accumulated fat mass rather than because EtOH‐treated mice lost fat. This distinction underscores that controls were not metabolically static; they continued to accrue adiposity over time, whereas EtOH‐treated mice reached a plateau. Accordingly, while previous studies have reported that EtOH enhances basal WAT lipolysis (Chen et al. 2009; Steiner and Lang 2017; Zhong et al. 2012), our observation that controls had higher free fatty acids in WAT may instead reflect greater lipid storage in control mice rather than enhanced lipolysis in EtOH‐treated mice. Nevertheless, our findings support the conclusion that systemic metabolic and physical dysfunction in EtOH‐treated mice is driven primarily by perturbations along the gut–liver axis (Raya Tonetti et al. 2024; Szabo 2015; Tripathi et al. 2018).

Several limitations should be acknowledged. First, the study included only female mice, which may overlook sex‐specific differences in EtOH susceptibility, as males can exhibit distinct microbiome and metabolic responses (da Brigagão Pacheco Silva et al. 2025; Chella Krishnan et al. 2018; de Souza et al. 2022; Kim et al. 2020; McGee and Huttenhower 2021). Second, although the long‐term exposure model improves translational relevance, constraints inherent to extended protocols yielded relatively small sample sizes and precluded housing mice individually and assessing intermediate time points for some variables. Third, the analysis focused primarily on the gut, liver, and WAT, potentially missing broader systemic effects, including those on the central nervous, digestive, and cardiovascular systems, constraints largely due to time and resource limitations. We refer readers to our previous work in skeletal muscle for related findings (Willis et al. 2026). Fourth, the absence of transcriptional changes in subcutaneous WAT does not necessarily indicate a lack of changes in protein content or activity, nor does it rule out EtOH‐induced effects in other fat depots (Zhang et al. 2015). Lastly, it is important to note that variability in preclinical EtOH models often stems from differences in dosing regimens and exposure duration. Despite these noted limitations, the present study provides critical insights into EtOH‐induced tissue injury and systemic dysfunction, offering a strong foundation for future mechanistic and translational research.

In conclusion, this study reveals a mechanistic cascade linking chronic EtOH exposure to gut–liver axis disruption and systemic metabolic dysfunction, with clear translational relevance. EtOH‐induced dysbiosis, characterized by depletion of *Lactobacillus*, enrichment of pro‐inflammatory taxa, and reduced SCFAs, compromised gut barrier integrity, increased permeability, and promoted endotoxemia. These changes coincided with hepatic injury, including membrane damage, inflammation, and amyloid deposition, positioning the liver as a central hub for EtOH toxicity. Interestingly, the enrichment of *Bifidobacterium*, an uncommon finding in alcohol studies, may represent a compensatory response to prolonged EtOH exposure and highlight microbiome‐targeted therapies as a promising intervention. Beyond gut and liver pathology, systemic effects such as dyslipidemia, glucose intolerance, and impaired physical performance, even under caloric restriction, underscore EtOH's impact on whole‐body physiology. Collectively, these findings advance our understanding of EtOH's multi‐organ toxicity and identify bile acid signaling and microbiome restoration as key therapeutic targets for alcoholic liver disease, while providing a robust preclinical platform for future mechanistic and translational studies.

## Author Contributions

Muni Swamy Ganjayi, Thomas A. Krauss, Gage E. Demster, Michael Tranter, Robert N. Helsley, and Cory W. Baumann conceived and designed the analysis. Muni Swamy Ganjayi, Thomas A. Krauss, Gage E. Demster, Sehyung Park, Garrett B. Anspach, Sarah R. Anthony, Michael Tranter, Robert N. Helsley, and Cory W. Baumann collected the data and performed the analysis. All authors contributed data or analysis tools. Muni Swamy Ganjayi and Cory W. Baumann wrote the paper. Muni Swamy Ganjayi, Michael Tranter, Robert N. Helsley, and Cory W. Baumann revised the paper. All authors contributed to the article and approved the submitted version.

## Funding

We acknowledge the support of the Osteopathic Heritage Foundation through funding for the Ralph S. Licklider, D.O., Endowed Faculty Fellowship (to Cory W. Baumann), Ohio University Heritage College of Osteopathic Medicine for Student Research Funding (to Gage E. Demster), the American Heart Association (23CDA1051959 to RNH), and the National Institutes of Health (R01DK139147 and K01DK128022 to RNH and R01HL158671 to MT).

## Ethics Statement

All procedures involving animals were approved by the Institutional Animal Care and Use Committee of the Ohio University, Athens, Ohio.

## Conflicts of Interest

The authors declare no conflicts of interest.

## Supporting information


**Table S1:** List of antibodies used.

## Data Availability

All data will be made available upon request.
