# Supplementary material for: Systemic Consequences of Chronic Ethanol Intake: From Microbiome Shifts to Metabolic Impairment
Source: Compr Physiol. 2026 Mar 26;16(2):e70132. doi: 10.1002/cph4.70132 (PMC13022470; doi:10.1002/cph4.70132)
Supplement: Supplementary file 1 — Table S1: List of antibodies used. [file CPH4-16-e70132-s001.docx]

| **Antibody** | **Host** | **Dilution** | **Catalogue/Company** |  |
| --- | --- | --- | --- | --- |
| MVK | Rabbit | 1:2000 | #12228-I-AP  Protein tech, Rosemont, IL USA | Incubation 1 Hr at RT or Overnight at 4°C |
| HMGCS | Rabbit | 1:2000 | 17643-I-AP  Protein tech, Rosemont, IL, USA |  |
| IL-6 | Rabbit | 1:3000 | #12153T  Cell Signaling, Denver, MA, USA |  |
| TNF-α | Rabbit | 1:3000 | #11948T  Cell Signaling, Denver, MA, USA |  |
| APP | Rabbit | 1:2000 | #25524-I-AP, Protein tech, Rosemont, IL, USA |  |
| LRP-1 | Rabbit | 1:2000 | # EPR3724, Abcam, Waltham, MA, USA |  |
| β-Actin | Mouse | 1:5000 | #SC-47778 Alexa Fluro 680 Conjugated  Santa Cruz, CA, USA |  |

**Supplementary Table 1: List of antibodies used.**
